# Patterns of Waterpipe and Cigarette Smoking Among Young People in Central Iran: A Population‐Based Cross‐Sectional Study

**DOI:** 10.1002/hsr2.73486

**Published:** 2026-11-05

**Authors:** Rahmatollah Moradzadeh, Javad Nazari, Irandokht Heidari, Faezeh Barati, Kiana Rezaei, Maryam Zamanian

**Affiliations:** ^1^ Department of Epidemiology, School of Health Arak University of Medical Sciences Arak Iran; ^2^ Department of Pediatrics, School of Medicine Arak university of Medical Sciences Arak Iran; ^3^ Deputy of Health Arak university of Medical Sciences Arak Iran

**Keywords:** Iran, smoking, waterpipe, young

## Abstract

**Background and Aims:**

Tobacco smoking and waterpipe use are common among Iranian youth, with notable gender differences influenced by cultural factors. This study aimed to assess patterns of cigarette and waterpipe consumption among young people in central Iran.

**Methods:**

This population‐based cross‐sectional study was conducted among 1384 young adults aged 18–30 years in urban and rural areas covered by Arak University of Medical Sciences. Participants were selected using multistage stratified cluster sampling. Data were collected via a structured self‐administered questionnaire assessing sociodemographic characteristics, tobacco use patterns (cigarette and waterpipe), exposure to secondhand smoke, and reasons for initiation and continuation of tobacco use. Independent *t*‐tests and Chi‐square tests or Fisher's exact test were applied, with *p* < 0.05 considered statistically significant.

**Results:**

Among 1384 participants (779 females and 605 males), current cigarette or waterpipe use was reported by 26.9% of males and 5.7% of females. In adjusted multinomial logistic regression, males had higher relative risk ratios for not‐daily (RRR = 2.02, 95% CI: 1.23–3.34) and daily use (RRR = 8.54, 95% CI: 2.70–27.03) compared with no use. Older age was associated with daily use (RRR = 1.13, 95% CI: 1.03–1.23). Cigarette initiation was more frequently reported among males than females, while waterpipe initiation showed a smaller gender difference. Males also reported greater secondhand smoke exposure and more recent cessation attempts.

**Conclusion:**

Tobacco use was more prevalent and socially motivated among males. Gender‐sensitive interventions targeting cultural norms and misconceptions about waterpipe smoking are recommended.

## 1 Introduction

Tobacco use remains one of the leading preventable causes of morbidity and mortality worldwide [1], contributing significantly to noncommunicable diseases, including cancer, cardiovascular disease, and chronic respiratory conditions. Despite substantial progress in tobacco control, the global burden remains considerable. According to the World Health Organization (WHO), approximately 1.2 billion people aged 15 years and older were current tobacco users in 2024, indicating that tobacco use continues to be a major global public health challenge [2]. Waterpipe smoking is an important form of tobacco use among young people, particularly in the Eastern Mediterranean Region (EMR) [3, 4].

EMR is of particular concern because it has one of the highest burdens of waterpipe tobacco use worldwide. Waterpipe smoking is especially common among young people in the region and, in several countries, is more prevalent than cigarette smoking among adolescents [5, 6]. According to WHO estimates, the EMR has the highest prevalence of waterpipe smoking among WHO regions, while tobacco use among adolescents remains a major public health concern [3]. The relatively high social acceptability of waterpipe smoking, together with misconceptions regarding its harmfulness and its association with social and recreational activities, may contribute to its widespread use among young people in the region [5, 6].

In Iran, the prevalence of tobacco use varies across age groups and types of products. The national data indicate that current tobacco smoking among adults remains substantial, with higher rates observed among males compared to females. According to the 2021 STEPS survey, approximately 14% of Iranian adults were current tobacco smokers, with a significant sex gap (approximately 25.9% in males vs. 4.4% in females), and waterpipe use was reported by 5.6% of males and 3.6% of females [7]. These estimates indicate that, alongside cigarette smoking, waterpipe use remains a relevant component of tobacco consumption in Iran. Previous population‐based studies in Tehran showed a prevalence of current waterpipe smoking of around 17.6%, with males almost twice as likely to use waterpipe as females [8]. Although the overall prevalence of cigarette smoking has shown possible stabilization in recent years, waterpipe smoking remains particularly common among younger adults and adolescents, reflecting shifting patterns of tobacco consumption in the Iranian context. Studies among adolescents in Tehran reported waterpipe use prevalence as high as 25.7%, with a greater prevalence in males than females, indicating both increasing uptake and gender disparities from early ages [9]. However, evidence describing tobacco‐use patterns at the subnational level, particularly in central Iran and Markazi Province, remains relatively limited. Provincial‐level studies are important because tobacco‐use patterns may vary according to local socioeconomic conditions, cultural norms, family structure, and social environments [10].

Gender differences in tobacco use behavior extend beyond prevalence rates and encompass patterns and motivations. Iranian and global studies consistently show that males are more likely to initiate tobacco use earlier and to use a broader range of tobacco products than females. A recent systematic review among Iranian students reported smoking prevalence of 26% in male students compared to 7% in female students, reflecting cultural and social norms that deter females from cigarette smoking but may exert less influence on waterpipe use [11]. Similarly, international evidence suggests that beliefs about the social acceptability of waterpipe smoking, perceptions of lower harm, and the social appeal of shared usage contribute to its popularity among both sexes, though the gender gap remains narrower for waterpipe than for cigarette smoking [8].

Socio‐economic and environmental determinants further complicate tobacco use patterns. Large cohort data from the PERSIAN study revealed that waterpipe smoking prevalence in Iran is influenced by age, sex, education, and lifestyle factors, with higher prevalence observed among males, younger adults, and those with higher socio‐economic status [12]. These findings indicate that tobacco use in Iran varies according to demographic and lifestyle characteristics, including sex, age, education, and socioeconomic status.

Despite the growing body of evidence on tobacco use among Iranian adolescents and young adults, several important gaps remain. First, previous studies have predominantly focused on the prevalence of tobacco use, while less attention has been given to differences between females and males in the broader pattern of tobacco consumption, including the type of product used at initiation, frequency of current use, secondhand smoke exposure, and attempts to quit. Second, cigarette and waterpipe use are often examined separately, limiting understanding of how gender may influence preferences for different tobacco products and the reasons for their initiation and continuation. Third, much of the available evidence is derived from large cities or national surveys, whereas population‐based evidence from central Iran, particularly Markazi Province, remains limited. Consequently, there is a need for population‐based studies that simultaneously examine cigarette and waterpipe use, gender‐specific patterns, and the social and behavioral factors associated with initiation and continuation among young people in this region. Therefore, this study aimed to address these gaps by examining gender‐specific patterns of cigarette and waterpipe use among young people in Markazi Province, central Iran. Specifically, we assessed differences in current and past tobacco use, type and age of initiation, secondhand smoke exposure, quit attempts, and the reported reasons for initiation and continuation of tobacco use.

## 2 Materials and Methods

### 2.1 Study Design and Sampling Procedure

This cross‐sectional study was conducted in Markazi Province, Iran, as part of the National Survey of Health and Risk Behaviors among Youth. The target population consisted of young adults aged 18–30 years residing in urban and rural areas covered by Arak University of Medical Sciences in the nine counties: Arak, Shazand, Delijan, Komijan, Mahallat, Farahan, Ashtian, Tafresh, and Khondab. These nine counties constituted the geographic strata of the sampling framework, and participants were selected from each stratum.

A multistage stratified cluster sampling method was employed. First, the required sample size for Markazi Province was determined according to the national survey protocol and based on the proportion of the youth population covered by the university.

The sample size was obtained according to the following formula:

$$n=\frac{Z_{1-\frac{\alpha }{2}}^{2}\times (p\left(1-p\right))}{d^{2}},$$

where *n* is the required sample size, the $Z_{1-\frac{\alpha }{2}}^{2}$ corresponding to a 95% confidence level is 1.96, *p* is the expected prevalence of the variable under study, and *d* is the precision of the estimate. Considering the estimated prevalence of smoking of 1.8% in the study of Zamanian et al. [10], as well as the *d* = 0.0075, the minimum sample size was calculated to be 1207. Because a larger number of eligible individuals was accessible within the study area, invitations were sent to 1647 eligible individuals. Of these, 263 declined to participate, resulting in an overall response rate of 84% and a final sample of 1384 participants. An approximately equal proportion of females and males was targeted within the urban and rural sampling strata during the sampling stage. However, the final analytical sample comprised 779 females (56.3%) and 605 males (43.7%). The planned 50:50 allocation was not used as a poststratification weighting target, and no weighting was applied to force the final sample to an equal gender distribution.

In the first stage, counties and areas covered by Arak University of Medical Sciences were selected. Subsequently, the required clusters were identified from urban and rural regions. Within each cluster, eligible households were selected using systematic sampling. A list of households containing individuals aged 18–30 years was prepared, and the sampling interval (*k*) was calculated by dividing the total number of eligible individuals by the required sample size. After randomly selecting a starting point, households were chosen at regular intervals until the target sample size was achieved. Eligible individuals within selected households were then randomly recruited into the study.

### 2.2 Data Collection Tool

Data were collected using the standardized questionnaire of the National Survey of Health and Risk Behaviors among Youth. The questionnaire was self‐administered in the presence of trained interviewers; however, for participants unable to complete the questionnaire independently, face‐to‐face interviews were conducted by the interviewers. Prior to data collection, the objectives of the study were explained to all participants, and they were assured of the confidentiality and anonymity of their responses. To ensure privacy, no identifying information, including names, telephone numbers, or addresses, was recorded on the questionnaires.

Before the commencement of data collection, interviewers received comprehensive training regarding study procedures, research ethics, participant communication, and proper questionnaire administration. The questionnaire was primarily self‐administered to enhance privacy when reporting sensitive behaviors. Trained interviewers provided instructions and assistance when required, and face‐to‐face administration was used for participants who were unable to complete the questionnaire independently. Participants were informed that their responses would be kept confidential and used only for research purposes. In addition, university and field supervisors continuously monitored the sampling process, data collection procedures, and questionnaire quality. The questionnaire consisted of three main sections:

### 2.3 Sociodemographic Characteristics

This section included questions on gender, marital status (single, married, divorced), living arrangement (dormitory, private house, family home, other), employment status (employed, unemployed, housewife), occupational or social status (student, soldier, seminary student, other), household composition (living with both parents, one parent, spouse and children, living alone, etc.), and educational level (primary, secondary, diploma, university degree). The purpose of this section was to describe participants’ background characteristics and to assess possible gender differences across these variables.

### 2.4 Tobacco Use Status and Exposure to Tobacco Smoke

The second section assessed tobacco use and exposure to tobacco smoke. Current tobacco use (cigarette or waterpipe) was categorized as daily use or not‐daily use. Daily use referred to current tobacco use on a daily basis, whereas not‐daily use referred to current tobacco use that was not daily. Participants who had ever used tobacco were also asked about their age at first use and the type of tobacco used at first experience (cigarette or waterpipe). Participants were additionally asked to report the number of cigarettes or waterpipe sessions consumed on a daily, weekly, and monthly basis.

The questionnaire also assessed whether participants had used tobacco on a daily basis in the past and whether they currently used smokeless tobacco products. Current use of smokeless tobacco was assessed using the question, “Do you currently use smokeless tobacco products (such as chewing tobacco and other similar products)?” Additional questions assessed secondhand smoke exposure at home and exposure to tobacco smoke in the workplace during the past 30 days, as well as any attempt to quit tobacco use during the previous year.

### 2.5 Reasons for Initiation and Continuation of Tobacco Use

In this section, participants who reported tobacco use were asked to indicate their main reasons for starting and continuing tobacco consumption. Response options included: reduction of anxiety, increased mental concentration, social acceptance by peers, feeling of pleasure, forgetting or escaping from problems, recreation and entertainment, belief in the harmlessness of tobacco use, feeling of self‐confidence, peer pressure, and other possible reasons. Participants could select more than one option. After initial design, the questionnaire was reviewed by a group of experts in community health and addiction behavior for content validity and revised accordingly. To assess reliability, a pilot study was conducted on 30 individuals with similar characteristics to the target population. Cronbach's alpha coefficients ranged from 0.78 to 0.85, indicating satisfactory internal consistency. The questionnaires were distributed anonymously and self‐administered among participants. After completion, responses were reviewed for completeness and entered into statistical software for analysis.

### 2.6 Statistical Analysis

Continuous variables were reported as mean ± standard deviation (Mean ± SD), and categorical variables were presented as frequency and percentage. For descriptive proportions, 95% confidence intervals (CIs) were calculated using the Wilson method. Percentages and 95% CIs were rounded to one decimal place. Independent *t*‐tests were used to compare means, and Chi‐square tests were applied to examine associations between categorical variables. When expected cell counts were too small for the Pearson chi‐square test, Fisher's exact test was used, where applicable. Statistical significance was defined as a two‐sided *p* < 0.05. The initial descriptive analyses were performed using SPSS version 23.0, whereas the revised multinomial logistic regression analysis was performed using Stata 18.0.

To assess factors associated with current tobacco use, a multinomial logistic regression model was fitted, with no current tobacco use as the reference outcome category. Current tobacco use was categorized as no use, not‐daily use, and daily use. Age was entered into the model as a continuous variable, while gender, marital status, educational level, and employment status were entered as categorical variables. Female participants and the reference categories shown in Table 2 were used as the respective reference groups. Adjusted relative risk ratios (RRRs) with 95% CIs and *p*‐values were reported. Statistical significance was defined as *p* < 0.05.

Because the present analysis was restricted to the population covered by Arak University of Medical Sciences and was not intended to generate nationally representative estimates, national survey weights were not applied. The nine sampling strata used for participant selection within the study area were described in the sampling procedure. The statistical analyses were conducted to examine gender‐specific differences within the study population.

Missing data were assessed for each study variable. Because the proportion of missing observations varied across variables, analyses were conducted using available observations for each variable. Participants with missing information for a specific variable were excluded only from the analysis of that variable and were retained in analyses of other variables for which information was available. The denominators for each variable were therefore reported explicitly in the tables.

## 3 Results

As shown in Table 1, there were statistically significant differences between males and females in marital status, living arrangement, employment status, occupational status, and household composition (*p* values are indicated in the Table 1). In contrast, educational level and age did not differ significantly between the two groups (*p* values are indicated in the Table 1). Overall, 68.4% of males were single compared with 33.7% of females, whereas 63.6% of females were married compared with 30.3% of males. Regarding employment, 55.4% of males were employed, while 61.2% of females were housewives. In terms of household composition, 64.5% of males lived with both parents, whereas 55.8% of females lived with a spouse and children.

**Table 1:** Demographic variables of the participants by gender*.

Variable	Category	Female *n* (%)	Male *n* (%)	Total *n* (%)	*p*‐value
Age	Mean (SD)	23.9 (3.6)	24.1 (3.7)	24.0 (3.6)	0.24
Marital status	Single	262 (33.7%)	411 (68.4%)	673 (48.8%)	<0.001
	Married	494 (63.6%)	182 (30.3%)	676 (49.1%)	
	Divorced	21 (2.7%)	8 (1.3%)	29 (2.1%)	
Living arrangement	Dormitory	14 (1.8%)	12 (2.0%)	26 (1.9%)	0.02
	Student house	2 (0.3%)	4 (0.7%)	6 (0.4%)	
	Family home	755 (97.7%)	563 (95.6%)	1318 (96.8%)	
	Other	2 (0.3%)	10 (1.7%)	12 (0.9%)	
Employment status	Employed	90 (12.4%)	313 (55.4%)	403 (31.2%)	<0.001
	Unemployed	192 (26.4%)	242 (42.8%)	434 (33.6%)	
	Housewife	445 (61.2%)	10 (1.8%)	455 (35.2%)	
Occupation/status	Student	154 (24.6%)	157 (33.1%)	311 (28.3%)	<0.001
	Soldier	1 (0.2%)	32 (6.7%)	33 (3.0%)	
	Seminary student	14 (2.2%)	10 (2.1%)	24 (2.2%)	
	Other	456 (73.0%)	276 (58.1%)	732 (66.5%)	
Household composition	Both parents	278 (36.7%)	382 (64.5%)	660 (48.9%)	<0.001
	One parent	30 (4.0%)	38 (6.4%)	68 (5.0%)	
	Spouse and child(ren)	423 (55.8%)	148 (25.0%)	571 (42.3%)	
	Single parent with one child	5 (0.7%)	6 (1.0%)	11 (0.8%)	
	Living alone	4 (0.5%)	7 (1.2%)	11 (0.8%)	
	Other	18 (2.4%)	11 (1.9%)	29 (2.1%)	
Educational level	Illiterate/primary	32 (4.1%)	22 (3.7%)	54 (3.9%)	0.51
	Secondary/high school	176 (22.7%)	156 (26.1%)	332 (24.2%)	
	Diploma	272 (35.1%)	198 (33.2%)	470 (34.2%)	
	University degree	296 (38.1%)	221 (37.0%)	517 (37.7%)	

* Percentages are based on the number of participants with available data for each variable; therefore, denominators may vary across variables due to missing responses.

In the adjusted multinomial logistic regression model, male sex was associated with higher odds of both not‐daily (RRR = 2.02, 95% CI: 1.23–3.34; *p* = 0.006) and daily tobacco use (RRR = 8.54, 95% CI: 2.70–27.03; *p* < 0.001) compared with no use. Older age was associated with daily tobacco use (RRR = 1.13, 95% CI: 1.03–1.23; *p* = 0.01). University education and housewife status were associated with lower RRRs for daily tobacco use, while divorced status was associated with a higher RRR (Table 2).

**Table 2:** Multinomial logistic regression analysis of factors associated with current tobacco use.

Variable	Not‐daily versus no use RRR (95% CI)	*p*‐value	Daily versus no use RRR (95% CI)	*p*‐value
Age, per year	1.05 (0.99–1.12)	0.11	1.13 (1.03–1.23)	0.01
Gender				
Female	Reference	—	Reference	—
Male	2.02 (1.23–3.34)	0.006	8.54 (2.70–27.03)	<0.001
Marital status				
Single	Reference	—	Reference	—
Married	0.60 (0.36–1.01)	0.06	0.94 (0.48–1.83)	0.85
Divorced	2.07 (0.63–6.82)	0.23	4.94 (1.03–23.61)	0.04
Educational level				
Illiterate/primary	Reference	—	Reference	—
Secondary/high school	0.79 (0.29–2.12)	0.64	0.95 (0.28–3.23)	0.93
Diploma	0.70 (0.26–1.84)	0.47	0.77 (0.23–2.61)	0.68
University degree	0.52 (0.20–1.37)	0.19	0.27 (0.08–0.96)	0.04
Employment status				
Employed	Reference	—	Reference	—
Unemployed	0.77 (0.49–1.21)	0.26	0.73 (0.40–1.33)	0.30
Housewife	0.27 (0.13–0.60)	<0.001	0.15 (0.03–0.88)	0.04

Abbreviations: CI, confidence interval; RRR, relative risk ratio.

Table 3 presents the gender‐specific patterns of tobacco use and exposure among participants. There were statistically significant differences between males and females in most tobacco‐related variables (p values are indicated in the Table 3). Current tobacco use (including cigarette and waterpipe) was reported by 26.9% of males (11.2% daily, 15.7% not daily) compared with only 5.7% of females (0.8% daily use, 4.9% not daily use) (*p* < 0.001). The mean age at first use did not differ significantly between genders (females: 18.1 ± 4.8 years, males: 17.6 ± 3.7 years, *p* = 0.49). Regarding the type of first tobacco used, 13.6% of males and only 0.4% of females initiated with cigarettes, while 6.8% of males and 2.1% of females began with waterpipe (*p* < 0.001). Daily tobacco use in the past was significantly higher among males (10.4%) than females (1.2%) (*p* < 0.001). Similarly, current use of smokeless tobacco was more common among males (1.7%) than females (0.3%) (*p* = 0.005). Exposure to secondhand smoke at home was reported more frequently by males (48.6%) than females (40.1%) (*p* < 0.001). Likewise, exposure to tobacco smoke at work in the past 30 days was significantly higher among males (40.8%) compared with females (15.9%) (*p* < 0.001). Attempts to quit smoking within the past year were also more common among males (12.9%) than females (1.8%) (*p* < 0.001). No significant differences were found in the mean number of daily or weekly waterpipe or cigarette use between genders (*p* values are indicated in the Table 3). Overall, these findings indicate that tobacco use and exposure were substantially higher among males, while the age at initiation and consumption patterns among current users did not differ significantly between genders.

**Table 3:** The gender‐specific patterns of tobacco use and exposure among participants*.

Variables	Categories	Female (*n* = 779), % (95% CI)	Male (*n* = 605), % (95% CI)	All (*n* = 1384), % (95% CI)	*p*‐value
Current tobacco use (cigarette or waterpipe)	Daily use	0.8 (0.4–1.7)	11.2 (9.0–14.0)	5.3 (4.3–6.7)	<0.001
	Not daily use	4.9 (3.6–6.6)	15.7 (13.0–18.8)	9.6 (8.2–11.3)	
	No current use	94.4 (92.5–95.8)	73.1 (69.4–76.4)	85.0 (83.1–86.8)	
Age at first use	Mean (SD)	18.1 (4.8)	17.6 (3.7)	17.7 (3.9)	0.49
Type in first use	Cigarette	0.4 (0.1–1.1)	13.6 (11.1–16.5)	6.1 (5.0–7.5)	<0.001
	Waterpipe	2.1 (1.3–3.3)	6.8 (5.0–9.1)	4.1 (3.2–5.3)	
Daily tobacco use in the past	Yes	1.2 (0.6–2.2)	10.4 (8.2–13.1)	5.2 (4.2–6.5)	<0.001
Current use of smokeless tobacco	Yes	0.3 (0.1–0.9)	1.7 (0.9–3.0)	0.9 (0.5–1.5)	0.005
*N* of daily cigarette	Mean (SD)	3.0 (1.0)	9.4 (6.5)	9.1 (6.5)	0.10
*N* of weekly cigarette	Mean (SD)	—	35.8 (40.0)	33.5 (39.5)	—
*N* of monthly cigarette	Mean (SD)	—	85.9 (160.8)	85.9 (160.8)	—
*N* of daily waterpipe	Mean (SD)	1.8 (0.9)	1.4 (0.6)	1.5 (0.7)	0.39
*N* of weekly waterpipe	Mean (SD)	1.8 (0.8)	2.4 (3.4)	2.3 (3.2)	0.69
*N* of monthly waterpipe	Mean (SD)	1.3 (0.6)	2.7 (4.0)	2.2 (3.3)	0.12
Secondhand smoke exposure at home	Daily use	8.8 (6.9–10.9)	13.9 (11.4–16.9)	11.0 (9.4–12.7)	<0.001
	Weekly use	4.7 (3.5–6.5)	6.6 (4.9–8.9)	5.6 (4.5–6.9)	
	Monthly use	2.8 (1.9–4.2)	4.5 (3.1–6.4)	3.5 (2.7–4.7)	
	Sometimes use	16.4 (14.0–19.2)	15.4 (12.7–18.5)	16.0 (14.1–18.0)	
	Never	59.9 (56.5–63.3)	51.4 (47.4–55.4)	56.2 (53.6–58.8)	
Secondhand smoke exposure at work in past 30 days	Yes	15.9 (13.5–18.7)	40.8 (37.0–44.8)	26.8 (24.5–29.2)	<0.001
Any attempt on smoking cessation in past 1 year	Yes	1.8 (1.1–3.0)	12.9 (10.5–15.8)	6.6 (5.5–8.1)	<0.001

* Values are presented as % (95% CI) unless otherwise indicated. 95% CIs for proportions were calculated using the Wilson method. Mean (SD) values are presented without confidence intervals.

Among participants with valid responses, recreation was the most commonly reported reason for tobacco initiation (75.3%) and continued use (71.6%). Recreation was more frequently reported among nondaily than daily users for both initiation (81.2% vs. 64.2%, *p* = 0.02) and continued use (77.6% vs. 58.1%, *p* = 0.019). In contrast, mental concentration was more frequently reported by daily users for both initiation (28.2% vs. 6.7%, *p* = 0.003) and continued use (26.3% vs. 6.9%, *p* = 0.008). Reduction of anxiety was also more common among daily users as a reason for continued use (45.9% vs. 15.9%, *p* ≤ 0.001) (Table 4).

**Table 4:** The reported reasons for tobacco initiation and continuation among participants by gender*.

Variables	Daily users, *n* (%) (95% CI)	Nondaily users, *n* (%) (95% CI)	Total, *n* (%) (95% CI)	*p*‐value
Reason of tobacco initiation				
Reduction of anxiety	14 (35.9) (22.7–51.6)	13 (20.3) (12.3–31.7)	27 (26.2) (18.7–35.5)	0.08
Increased mental concentration	11 (28.2) (16.5–43.8)	4 (6.7) (2.6–15.9)	15 (15.2) (9.4–23.5)	0.003
Social acceptance by peers	10 (26.3) (15.0–42.0)	15 (22.1) (13.8–33.3)	25 (23.6) (16.5–32.5)	0.62
Feeling of being loved	2 (6.1) (1.7–19.6)	3 (5.0) (1.7–13.7)	5 (5.4) (2.3–12.0)	>0.99^a^
Enjoyment of the smell of tobacco	10 (26.3) (15.0–42.0)	7 (11.5) (5.7–21.8)	17 (17.2) (11.0–25.8)	0.06
Forgetting or escaping from problems	15 (37.5) (24.2–53.0)	18 (26.9) (17.7–38.5)	33 (30.8) (22.9–40.1)	0.25
Feeling of pleasure	13 (32.5) (20.1–48.0)	23 (33.3) (23.4–45.1)	36 (33.0) (24.9–42.3)	0.93
Recreation and entertainment	34 (64.2) (50.7–75.7)	82 (81.2) (72.5–87.6)	116 (75.3) (68.0–81.5)	0.02
Belief in the harmlessness of tobacco use	7 (19.4) (9.8–35.0)	5 (8.1) (3.5–17.5)	12 (12.2) (7.1–20.2)	0.12^a^
Peer pressure	2 (5.9) (1.6–19.1)	4 (6.9) (2.7–16.4)	6 (6.5) (3.0–13.5)	>0.99^a^
Feeling of self‐confidence	2 (5.9) (1.6–19.1)	3 (5.1) (1.7–13.9)	5 (5.4) (2.3–12.0)	>0.99^a^
Others	8 (22.2) (11.7–38.1)	8 (12.9) (6.7–23.4)	16 (16.3) (10.3–24.9)	0.23
Reason of tobacco use continuous				
Reduction of anxiety	17 (45.9) (31.0–61.6)	10 (15.9) (8.9–26.8)	27 (27.0) (19.3–36.4)	<0.001
Increased mental concentration	10 (26.3) (15.0–42.0)	4 (6.9) (2.7–16.4)	14 (14.6) (8.9–23.0)	0.008
Social acceptance by peers	9 (25.7) (14.2–42.1)	11 (16.9) (9.7–27.8)	20 (20.0) (13.3–28.9)	0.30
Feeling of being loved	2 (6.5) (1.8–20.7)	1 (1.8) (0.3–9.3)	3 (3.4) (1.2–9.5)	0.28^a^
Enjoyment of the smell of tobacco	7 (19.4) (9.8–35.0)	6 (10.2) (4.7–20.5)	13 (13.7) (8.2–22.0)	0.23^a^
Forgetting or escaping from problems	17 (44.7) (30.1–60.3)	18 (28.6) (18.9–40.7)	35 (34.7) (26.1–44.3)	0.01
Feeling of pleasure	16 (39.0) (25.7–54.3)	21 (32.3) (22.2–44.4)	37 (34.9) (26.5–44.4)	0.48
Recreation and entertainment	25 (58.1) (43.3–71.6)	76 (77.6) (68.3–84.7)	101 (71.6) (63.7–78.4)	0.02
Belief in the harmlessness of tobacco use	3 (9.4) (3.2–24.2)	3 (5.1) (1.7–13.9)	6 (6.6) (3.1–13.6)	0.66^a^
Peer pressure	3 (10.0) (3.5–25.6)	2 (3.6) (1.0–12.1)	5 (5.8) (2.5–12.9)	0.34^a^
Feeling of self‐confidence	2 (6.5) (1.8–20.7)	1 (1.8) (0.3–9.4)	3 (3.4) (1.2–9.7)	0.29^a^
Others	8 (23.5) (12.4–40.0)	8 (13.3) (6.9–24.2)	16 (17.0) (10.8–25.9)	0.21

* Values are presented as *n* (%) with 95% confidence intervals (CIs). Percentages for the daily and nondaily columns were calculated within each tobacco‐use‐frequency group. Total percentages were calculated among all participants with valid responses to each individual item. Therefore, the denominator varies across items. Missing responses were excluded from the corresponding analysis. The 95% CIs were calculated using the Wilson score method. Pearson's chi‐square test was used when expected cell counts were adequate; Fisher's exact test was used when one or more expected cell counts were less than 5 (^a^ Fisher's exact test).

## 4 Discussion

The results of this study demonstrate important differences in tobacco‐use patterns between males and females in Markazi Province, Iran. Current tobacco use was substantially more common among males, and this gender difference persisted after adjustment for age, marital status, educational level, and employment status. In the adjusted analysis, male participants had higher RRRs for both not‐daily and daily tobacco use compared with females, with a particularly pronounced association for daily use. These findings indicate that the observed gender disparity was not fully explained by the sociodemographic characteristics included in the model. Beyond prevalence differences, our findings also demonstrate gender‐specific patterns in the type of tobacco product used at initiation and in perceptions and reasons related to tobacco use.

The prevalence of current tobacco use was approximately 26.9% among males compared with 5.7% among females. The type of tobacco product used at initiation also differed by sex: cigarettes were more frequently reported as the first tobacco product among males, whereas waterpipe was more common among females. This pattern is consistent with previous evidence from Iran and other settings showing greater cigarette use among young males, while waterpipe use may exhibit a smaller gender gap. In a community‐based study among adolescents in Tehran, current waterpipe smoking was more prevalent among males than females (34.8% vs. 21.4%), although social factors such as having friends or family members who smoke were also associated with tobacco use [9, 13]. Similarly, national data from Iranian adults have shown substantially higher tobacco use among males, while waterpipe smoking has been reported more frequently among females than cigarette smoking [12]. These findings suggest that tobacco‐use patterns among young people are shaped not only by gender but also by the specific tobacco product being considered.

The findings of the present study regarding gender differences in the prevalence of tobacco use are consistent with the results of the study by Zamanian et al. [10], who found a substantial gender difference in cigarette and waterpipe smoking. The discrepancy between direct and indirect network scale‐up estimates in that study, particularly among females, may indicate the possibility of underreporting associated with the social sensitivity of tobacco use [10, 14]. This possibility is relevant when interpreting the lower prevalence observed among females in the present study. At the same time, the relatively frequent use of waterpipe reported among younger participants in previous research from Arak is consistent with our finding that waterpipe was more commonly reported as the first tobacco product among females [10]. A global review covering 114 countries similarly found that males were generally more likely than females to use tobacco products, including cigarettes and waterpipe [15].

Evidence from the Eastern Mediterranean region and the CASPIAN‐IV study in Iran has also documented higher cigarette use among young males, with gender differences varying according to the type of tobacco product [16, 17]. Additionally, a study conducted in Yazd reported that high‐risk behaviors, including tobacco use, were more prevalent among males than females, highlighting the interaction of social factors, parental supervision, and peer pressure in shaping consumption patterns [18].

Recreation was the most commonly reported reason for tobacco initiation and continued use, particularly among nondaily users, whereas daily users more often reported mental concentration and anxiety reduction. These findings suggest that perceived motives for tobacco use may differ by use frequency and should be considered in prevention and cessation interventions. Previous studies among young people have identified recreation, entertainment, stress relief, curiosity, and social interaction as common motives for waterpipe use, while perceptions that waterpipe is less harmful than cigarettes may contribute to its continued use [19].

The pattern of waterpipe use observed in the present study is also consistent with findings from the Tehran Cohort Study. Shafiee et al. [4], reported a weighted prevalence of current tobacco use of 19.8% in Tehran, with waterpipe accounting for a substantial proportion of tobacco consumption. Their findings indicated that waterpipe use occurred at younger ages and showed a smaller gender gap than cigarette smoking [4]. This pattern may help explain why waterpipe appeared more frequently than cigarettes as the first tobacco product among females in our study. Rather than treating waterpipe use simply as another form of tobacco consumption, these findings highlight the importance of addressing perceptions of its harmfulness and its social and recreational context in prevention programs.

The present findings have several implications for tobacco control among young adults. Prevention strategies should recognize the marked gender difference in tobacco use while also considering differences in the types of products used and the perceptions surrounding them. Particular attention should be given to correcting misconceptions regarding the harmfulness of waterpipe smoking and to addressing the motives associated with initiation and continuation. At the same time, the persistence of the gender association after adjustment for key sociodemographic characteristics suggests that prevention strategies should not rely solely on differences in marital status, education, or employment when designing interventions.

This study has several limitations. First, because of its cross‐sectional design, temporal and causal relationships between tobacco use and the examined characteristics cannot be established [20]. Second, tobacco use was assessed by self‐report, and underreporting cannot be excluded, particularly given the social sensitivity of tobacco use among females [21]. Finally, the study was conducted in a limited geographic area, and caution is therefore required when generalizing the findings to other regions of Iran.

## 5 Conclusion

Overall, this study found a substantially higher frequency of current tobacco use among males than females, and this gender difference persisted after adjustment for age, marital status, educational level, and employment status. Gender differences were also observed in the type of tobacco product used at initiation, with cigarettes more frequently reported among males and waterpipe more frequently reported among females. Some differences were also observed in the reasons and perceptions related to tobacco initiation and continuation. These findings highlight the importance of considering gender and tobacco‐product‐specific patterns when designing tobacco prevention and control strategies for young adults. In particular, prevention efforts should address misconceptions regarding the harmfulness of waterpipe smoking and the motivations and perceptions associated with tobacco initiation and continued use.

## Author Contributions

**Rahmatollah Moradzadeh:** conceptualization, investigation, Funding acquisition, writing – original draft, writing – review and editing, visualization, validation, methodology, software, formal analysis, project administration, supervision, data curation. **Javad Nazari:** conceptualization, writing – review and editing, formal analysis, validation, investigation, writing – original draft. **Irandokht Heidari:** validation, visualization, project administration; supervision, writing – review and editing, writing – original draft. **Faezeh Barati:** validation, supervision, project administration, visualization, writing – review and editing, writing – original draft, data curation. **Kiana Rezaei:** validation, visualization, writing – review and editing, writing – original draft, formal analysis, supervision, data curation. **Maryam Zamanian:** conceptualization, investigation, funding acquisition, writing – original draft, writing – review and editing, visualization, validation, methodology, software, formal analysis, project administration, data curation, supervision.

## Ethics Statement

The study was conducted in accordance with the Declaration of Helsinki and approved by the Ethics Committee of Arak University of Medical Sciences, Arak, Iran (Ethic approval code: IR.ARAKMU.REC.1405.233).

## Consent

Informed consent was obtained from all subjects involved in the study.

## Conflicts of Interest

The authors declare no conflict of interest.

## AI Disclosure

The authors used an artificial intelligence (AI)‐assisted tool, ChatGPT (OpenAI), for language editing.

## Transparency Statement

Maryam Zamanian affirms that this manuscript is an honest, accurate, and transparent account of the study being reported; that no important aspects of the study have been omitted; and that any discrepancies from the study as planned have been explained.

## Data Availability

The data presented in this study are available from the corresponding author upon reasonable request. All authors have read and approved the final version of the manuscript. Maryam Zamanian had full access to all of the data in this study and takes complete responsibility for the integrity of the data and the accuracy of the data analysis.
